# Grain Size Effect of the γ Phase Precipitation on Martensitic Transformation and Mechanical Properties of Ni–Mn–Sn–Fe Heusler Alloys

**DOI:** 10.3390/ma14092339

**Published:** 2021-04-30

**Authors:** Jinpei Guo, Minting Zhong, Wei Zhou, Yajiu Zhang, Zhigang Wu, Yingchao Li, Junsong Zhang, Yinong Liu, Hong Yang

**Affiliations:** 1 School of Civil Engineering, Guangzhou University, Guangzhou 510006, China; 2111816002@e.gzhu.edu.cn (J.G.); 2111916048@e.gzhu.edu.cn (M.Z.); zwdavid95@163.com (W.Z.); yjzh@gzhu.edu.cn (Y.Z.); 2 Department of Mechanical Engineering, The University of Western Australia, Perth, WA 6009, Australia; yingchao.li@research.uwa.edu.au (Y.L.); junsong.zhang@uwa.edu.au (J.Z.); yinong.liu@uwa.edu.au (Y.L.); hong.yang@uwa.edu.au (H.Y.)

**Keywords:** Heusler alloys, grain size, eutectic microstructure, martensitic transformation, mechanical properties, γ phase

## Abstract

Isothermal annealing of a eutectic dual phase Ni–Mn–Sn–Fe alloy was carried out to encourage grain growth and investigate the effects of grain size of the γ phase on the martensitic transformation behaviour and mechanical properties of the alloy. It is found that with the increase of the annealing time, the grain size and volume fraction of the γ phase both increased with the annealing time predominantly by the inter-diffusion of Fe and Sn elements between the γ phase and the Heusler matrix. The isothermal anneals resulted in the decrease of the e/a ratio and suppression of the martensitic transformation of the matrix phase. The fine γ phase microstructure with an average grain size of 0.31 μm showed higher fracture strength and ductility values by 28% and 77% compared to the coarse-grained counterpart with an average grain size of 3.31 μm. The fine dual phase microstructure shows a quasi-linear superelasticity of 4.2% and very small stress hysteresis during cyclic loading, while the coarse dual phase counterpart presents degraded superelasticity of 2.6% and large stress hysteresis. These findings indicate that grain size refinement of the γ phase is an effective approach in improving the mechanical and transformation properties of dual phase Heusler alloys.

## 1. Introduction

Ni–Mn-based Heusler alloys offer multiple useful properties as functional materials, such as the magnetic-field-induced shape memory effect [1,2,3] and giant magnetocaloric effect [4,5,6,7]. As a result, they have attracted much attention and have been extensively studied. Over the past decade, new alloy compositions and novel fabrication techniques have been developed, leading to much improved mechanical properties and discovery of new functional properties. For example, large elastocaloric effects have been realized in these alloys recently. An 8 K elastocaloric temperature change induced by 1.3% transformation strain was recorded of a Ni_44_Mn_41_Sn_11_Cu_4_ alloy [8] and a temperature change of 11.5 K was achieved in an all-d-metal Ni_35.5_Co_14.5_Mn_35_Ti_15_ alloy with a low critical stress of 38 MPa [9]. Ultra-large tensile superelastic strains of 14.0% and 20.0% were achieved in Ni_52.87_Mn_23.82_Ga_23.32_ [10] and Ni_50_Mn_31.4_Sn_9.6_Fe_9_ microwires [11] fabricated by the Taylor–Ulitovsky method. The achievements of such novel properties have motivated active research on the Ni-Mn-based alloy system in the past two decades.

However, these alloys also face some challenges. Their novel functionalities are only of benefit if their mechanical properties are satisfactory. This is the reason why good shape memory effect and superelasticity are only achieved in either single crystals [12,13] or low dimensional textured polycrystals [14,15], in which reasonable strength and ductility can be guaranteed. It is well known that obtaining good shape memory properties is still challenging in bulk polycrystalline Heusler alloys due to the grain boundary brittleness. The vulnerable mechanical properties of polycrystalline Heusler alloys also make them impractical for conventional material processing, thus severely limiting their applications.

Introducing a ductile second phase, e.g., a disordered fcc γ phase, is a common approach to improving the ductility of these alloys. The purpose of introducing the ductile γ phase is to interact or interfere with the progression of cleavage cracks in the brittle Heusler matrix. This is usually achieved by adding a fourth element such as Fe [16], Co [17,18], Cr [19], Cu [20] or extra Ni [21] in Ni–Mn–X (X = Ga, In, Sn) Heusler alloys. It is known that the γ phase does not participate in the martensitic transformation, and hence its presence in the Heusler matrix hinders the activities of the martensite variants and compromises the shape memory properties. Yang et al. reported that the shape memory recovery strain decreased with increasing amount of the γ phase and the shape memory effect almost diminished when the γ phase is over 45% in volume fraction in dual phase Ni–Mn–Ga alloys [22,23]. This suggests that the volume fraction of the γ phase shall be limited despite its positive effect on ductility. They also found that thermal cycling stability and shape memory effect of Ni–Mn–Ga–Fe were the best among Ni/Cu/Co/Fe doped Ni–Mn–Ga systems because the strength of the γ phase in Fe-doped system was the lowest, thus presenting the smallest resistance to martensitic transformation [23]. This suggests that the Fe-doped dual phase Ni–Mn–based alloys may be promising for practical use if an optimized combination of ductility and shape memory effect is achieved.

In addition to the volume fraction, the morphology of the γ phase also plays an important role in toughening the alloys, including the grain size, orientation and distribution. Villa et al. studied the γ phase distribution correlation with improvements in mechanical and functional properties. They found that the localization of the γ phase at the grain boundaries has the maximum benefits for ductility improvement and the least adverse effect on shape memory effect and pseudoelasticity in a Ni–Mn–Ga-Fe system [24]. Grain size is another important aspect which affects toughening mechanism of the γ phase, which has been largely overlooked in the literature. The reported γ phase normally presents a coarse grain morphology with an average grain size of tens of microns [22,25,26], though the random orientation and uniform dispersion are often available. The large grain size of the γ phase is the result of the solution treatment carried out at high temperatures over long periods necessary to eliminate the compositional segregation from coring of the Heusler matrix in the cast ingots. Due to its brittleness, grain refinement of the γ phase by deformation and recrystallization is practically impossible in these alloys. In our previous reports, a new grain refining strategy of the γ phase was introduced using the eutectic solidification method with a proper selection of alloy compositions, as demonstrated in Ni–Mn–Sn–Fe alloys [27,28]. The ultrafine eutectic lamellae represents a typical isotropic fine grained microstructure that exhibited high strength and ductility. 

In this work, we carried out a series of isothermal anneals for the fine eutectic alloy to obtain various microstructures with enlarged grains of the γ phase. This provides an opportunity to clarify the effects of the grain size of the γ phase on the martensitic transformation behaviour and mechanical properties of these dual phase Heusler alloys.

## 2. Materials and Methods

A polycrystalline Ni_51.5_Mn_34_Fe_6_Sn_8.5_ ingot of ~100 g was prepared by means of arc melting in argon atmosphere using high purity (99.95 wt%) elemental metals. The ingot was sectioned into 3 × 3 × 5 mm^3^ blocks for the compression test, ~5 × 10 × 2 mm^3^ for the micrographic examination and 10–20 mg (irregular shape) for the DSC measurement by means of spark wire cutting. The samples were annealed at 950 °C for different time durations (1, 2, 4, 8, 16, 32 and 120 h) in vacuum in a quartz tube furnace followed by water quench at the room temperature of 25 °C. The samples are designated as As-cast, A1, A2, A4, A8, A16, A32 and A120 according to the different annealing times. Microstructure and chemical analysis of the phases were examined using an FEI Verios 460 scanning electron microscope (SEM) equipped with an X-ray energy dispersive spectrometry (EDS) detector (ThermoFisher Scientific, Hillsboro, OR, USA). The metallographic samples were polished successively using abrasive papers with different grit numbers (120#, 240#, 500#, 1000#) and polishing cloth with diamond suspensions (6 μm, 3 μm, 1 μm). The polished surface was then examined by SEM without etching. Phase volume fractions and grain sizes in each alloy were estimated based on image analysis of the back-scattered SEM micrographs using an Image J software (an open platform software by National Institute of Health, Stapleton, NY, USA). The average grain size of the γ phase was estimated using the intercept method across the thickness of the grains on SEM micrographs at 5000 X comprising of at least 100 grains. Transformation behaviour of the alloys was analysed by means of differential scanning calorimetry (DSC) using a TA DSC 250 (TA Instrument, New Castle, DE, USA) with a cooling/heating rate of 10 K/min. Compression tests were conducted using an Instron 5982 universal testing machine (Instron Limited, Norwood, MA, USA) with a strain rate of 10^−4^/s at room temperature. Five compression measurements were conducted for each data.

## 3. Results and Discussion

### 3.1. Microstructure and Composition Analysis

**Figure 1:**
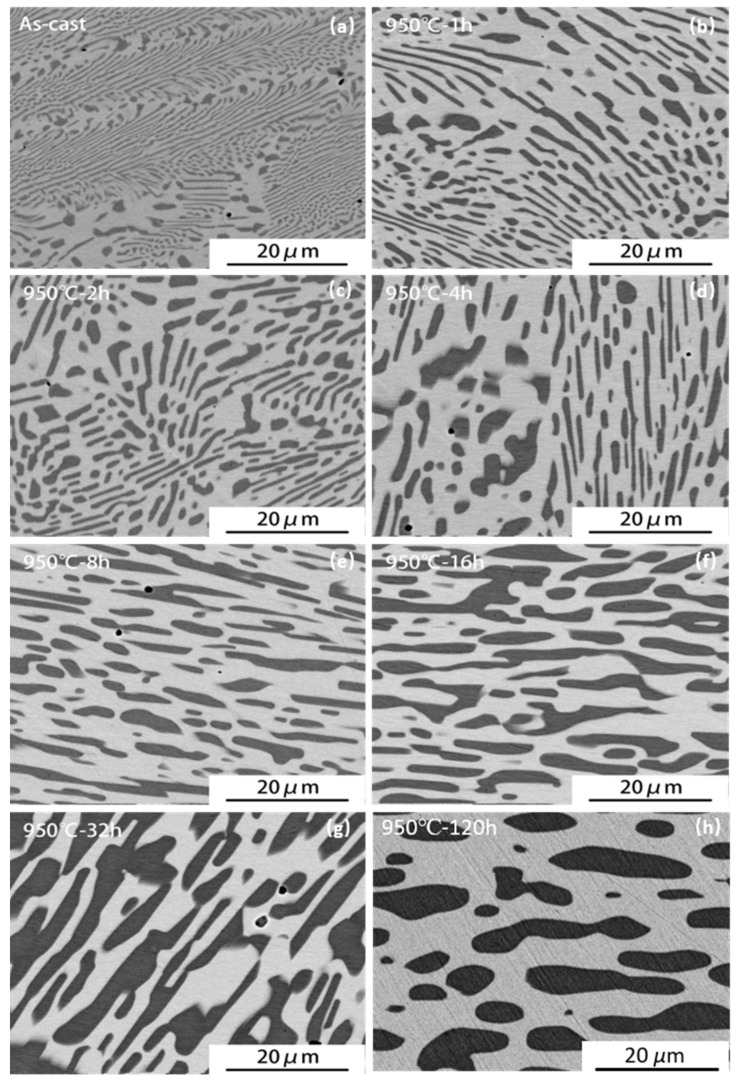
Back-scattered SEM micrographs of the Ni_51.5_Mn_34_Fe_6_Sn_8.5_ alloy after annealing for different durations: (**a**) As-cast, (**b**) A1, (**c**) A2, (**d**) A4, (**e**) A8, (**f**) A16, (**g**) A32 and (**h**) A120. The dark phase is the γ phase and the light phase is the Heusler phase.

Figure 1 shows the back-scattered SEM micrographs of the as-cast and annealed samples. It is seen that the As-cast sample has a fine lamellar dual phase microstructure of a Heusler matrix (light contrast) and γ phase lamellae (dark contrast) as shown in micrograph (a). The fine lamellar microstructure is resulted from a eutectic reaction during solidification as confirmed by the high temperature DSC measurement (not shown here). Annealing at 950 °C caused a significant and progressive coarsening of the eutectic lamellae with increasing the annealing duration, apparently due to the Oswald ripening effect [29,30] (micrographs (b)–(h)). The characteristic of the layered lamellar structure is hardly recognizable after annealing for 4 h. The average thickness of the γ phase grains increased from ~0.31 μm in the as-cast state to ~3.31 μm after 120 h of annealing at 950 °C (Table 1). 

**Figure 2:**
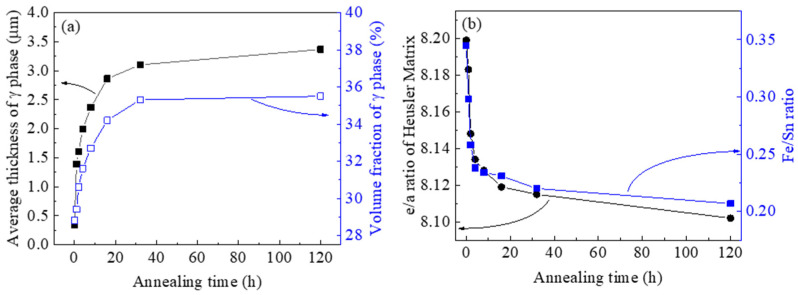
Effect of annealing time on (**a**) the average thickness and volume fraction of γ phase and (**b**) the e/a ratio and Fe/Sn ratio of the Heusler matrix.

Figure 2 shows the effects of annealing time on the γ phase morphology and the matrix phase composition. Figure 2a shows the evolution of the average thickness of γ phase lamellae as a function of annealing time. It is seen that the grain size increases rapidly initially before 20 h of heating and then the growth rate appears to be rather slow and eventually stabilized under 3.5 μm with prolonged holding time. The relatively slow grain growth may be due to the solute drag effects in the quaternary system [31,32] and the pinning effects from the dual phase interfaces [33,34] that act against the grain growth direction.

Additionally, plotted in Figure 2a is the evolution of the volume fraction of the γ phase. It increased from 28.8% to 35.5% with increasing the annealing time. The change in the volume fractions implies the alteration of the composition of each phase. The elemental concentrations of the phases were determined by EDS and the results are given in Table 1. The Heusler matrix is practically a Ni–Mn–Sn phase with a small amount of Fe in a solid solution, whereas the γ phase is a Ni–Mn–Fe phase containing a small amount of Sn. Annealing at 950 °C caused a progressive increase of the Sn content and decrease of the Fe content in the Heusler phase. This indicates that the As-cast structure is not at compositional equilibrium due to the fast-cooling rate of the solidification.

The e/a ratio of the Heusler phase was calculated using the sum of *s*, *p* and *d* electrons for Ni (10), Mn (7), Fe (8) and Sn (4) based on the chemical compositions and plotted against the annealing time in Figure 2b. The Fe/Sn ratio of the Heusler phase is also shown in the figure. It is seen that the e/a ratio decreased with the increase of annealing time. This is obviously a direct result of the decrease of Fe/Sn ratio with annealing time. The decrease of e/a ratio of the Heusler matrix phase implies a decrease of the martensitic transformation temperatures [35].

### 3.2. Phase Structure and Transformation Behaviour

**Figure 3:**
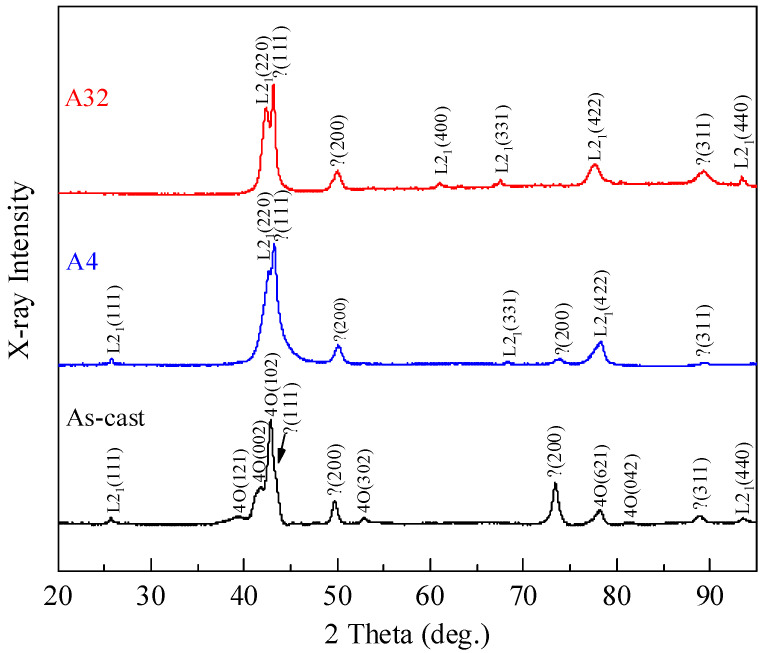
XRD spectra of As-cast, A4 and A32 samples at room temperature.

Figure 3 shows the XRD spectra of As-cast, A4 and A32 samples measured at room temperature. The diffraction peaks of each phase of the samples are indexed according to the literature [27,28,36,37,38]. It is seen that the As-cast sample contains mixed phases of L2_1_ austenite, 4O martensite and fcc γ phase at room temperature. Following annealing at 950 °C for 4 and 32 h, the samples showed only the diffraction peaks of L2_1_ austenite and fcc γ phase, while the diffraction peaks of the 4O martensite diminished. This suggests that the martensite transformation is a L2_1_↔4O type and the prolonged annealing has lowered the martensitic transformation temperatures of the alloys.

**Figure 4:**
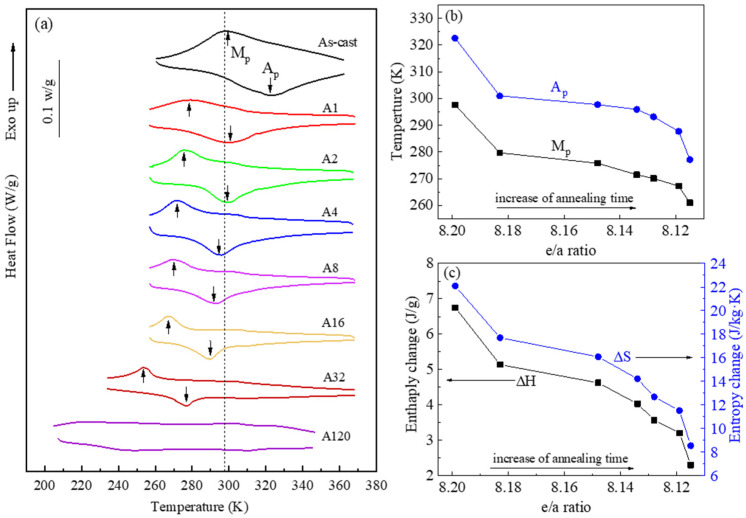
Martensitic transformation behaviour of Ni_51.5_Mn_34_Fe_6_Sn_8.5_ alloys with different annealing conditions. (**a**) DSC curves, (**b**) Effect of annealing time on martensitic transformation peak temperatures Mp and Ap and (**c**) Effect of annealing time on transformation enthalpy and entropy changes. The dashed vertical line in (**a**) indicates the testing temperature at 298 K.

Figure 4 shows the evolution of martensitic transformation with annealing time. Figure 4a presents the DSC curves of the As-cast and A1–A32 samples, which exhibited the L2_1_↔4O transformation peaks [28]. The A120 sample did not show any transformation peaks on the DSC curve within the temperature range tested. It is seen that As-cast and A1 have a mixed state of the austenite and martensite while A2–A32 samples mainly have the austenitic phase at room temperature (the dashed vertical line). The peak temperatures of the forward transformation (M_p_) and reverse transformation (A_p_) were plotted against the e/a ratio in Figure 4b. Both temperatures decreased with increasing annealing time, which is obviously due to the decrease in e/a ratio in the Heusler phase (Table 1). Figure 4c shows the enthalpy change and entropy change of the L2_1_↔4O transformation as functions of the e/a ratio. The enthalpy change is determined from the DSC curves and corrected for the Heusler phase only (ΔH) based on its volume fraction in each sample. The entropy change is estimated based on $\Delta S=\frac{\Delta H}{T_{0}}$, where $T_{0}=\frac{M_{p}+A_{p}}{2}$. It is seen that both ΔH and ΔS decreased with increasing the e/a ratio of the Heusler matrix. In this case, since isothermal annealing has changed the composition and effectively created different alloys among the samples, thus resulting in the variation of ΔS.

### 3.3. Thermomagnetic Behaviour

**Figure 5:**
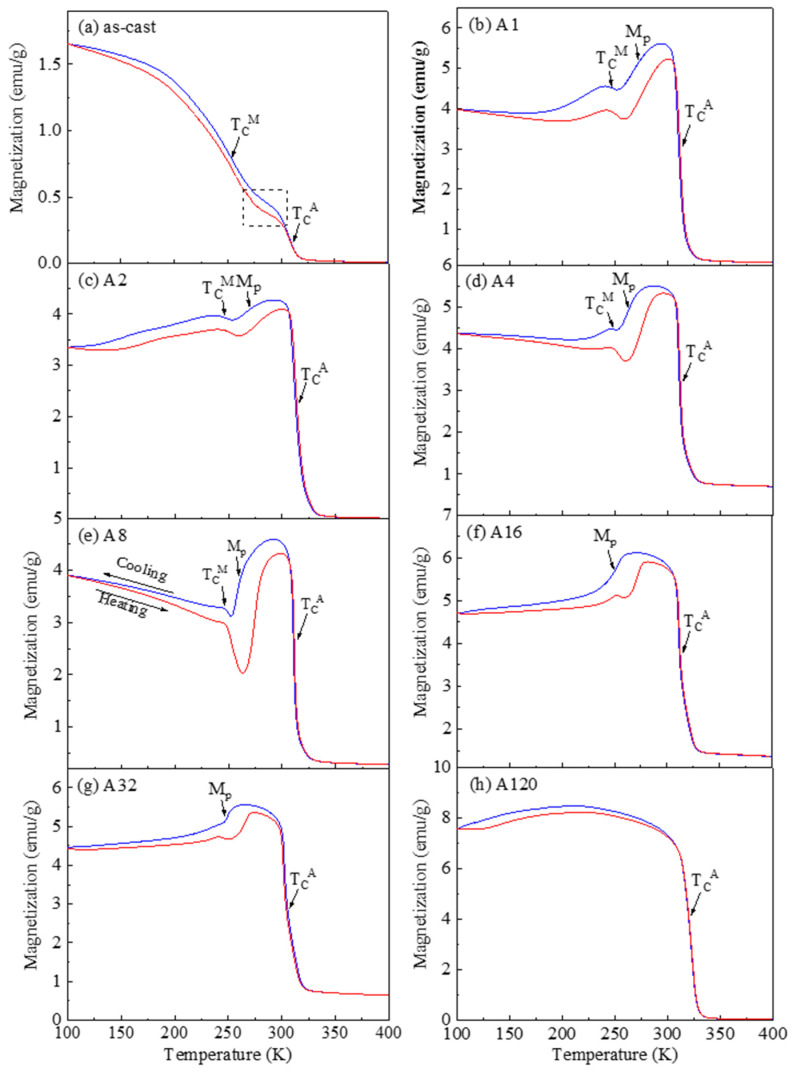
Thermomagnetic behaviour of the samples. (**a**) As-cast, (**b**) A1, (**c**) A2, (**d**) A4, (**e**) A8, (**f**) A16, (**g**) A32 and (**h**) A120. The blue and red curves represent the cooling and heating processes, respectively.

Figure 5 shows the thermomagnetic behaviour of the as-cast and annealed samples in the temperature range of 100–400 K. The blue and red curves represent the cooling and heating processes, respectively. Figure 5a shows the M-T response of the As-cast sample, which shows very low magnetization values within the tested temperature range. Upon cooling, the magnetization is nearly zero until a sharp rise near 300 K, corresponding to the Curie transition of the austenite, i.e., $T_{C}^{A}$. The rate of the magnetization gain decreased between 270 K and 300 K (dashed box), which may be due to the continued progression of martensitic transformation, being consistent with the DSC observation in Figure 4. Further cooling has caused the rapid increase of magnetization again due to the Curie transition of the martensite, i.e., $T_{C}^{M}$, followed by the saturation at around 1.65 emu/g at 100 K. The heating curve almost repeated the cooling curve, except for the section between 270 K and 300 K, in which an apparent thermal hysteresis appeared because of the martensitic transformation. The result indicates that the As-cast sample does not have the metamagnetic transformation characteristic due to lacking the abrupt magnetization change across the martensitic transformation.

Figure 5b–g show a similar thermomagnetic behaviour. Upon cooling, the $T_{C}^{A}$ appears as a sharp magnetization rise at 300–310 K, followed by a metamagnetic martensitic transformation where magnetization drops, and then the $T_{C}^{M}$ appears as the second magnetization rise at around 250 K. The $T_{C}^{A}$ and $T_{C}^{M}$ appear to be independent on the annealing time, while the $M_{p}$ (the temperature where the magnetization change is the steepest) decreased with increasing the annealing time. It is noted that A120 did not present a martensitic transformation even when the temperature was lowered to 100 K.

### 3.4. Mechanical Properties and Superelasticity

**Figure 6:**
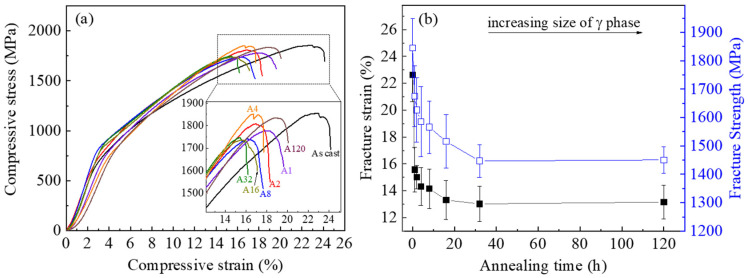
Mechanical properties of the alloys at 298 K. (**a**) Compressive stress–strain curves. (**b**) Effect of annealing time on fracture strain and fracture strength. Inset in (**a**) shows the enlarged view of the stress–strain curves near the fracture point labelled with sample names.

Figure 6a shows the mechanical properties of the samples. The As-cast sample showed the highest ductility (inset in (a)), though it has the smallest volume fraction of the γ phase among all samples. The annealed samples showed small variations in yield strength and fracture strength. Figure 6b plots the fracture strength and strain values that were normalized as per the same amount of volume fraction of the γ phase in the As-cast state (28.8%) for all samples. This reveals that both the fracture strength and strain decreased monotonically with the increase of annealing time, i.e., the increase of the grain size of the γ phase. The fracture strength decreased from about 1845 MPa to 1447 MPa, and the fracture strain decreased from about 23% to 13%, with increasing the grain size of γ phase by 10 folds. This means the fine-grained structure is 28% and 77% more superior in strength and ductility relative to the coarse-grained counterpart given the same amount of γ phase. It shall also be noted that the much-coarsened A120 sample did not show any martensitic transformation even at low temperatures (down to 100 K). This implies that the samples with γ phase grain size greater than 3.0 μm may not be applicable, not only due to the degraded mechanical properties but also because of the loss of martensitic transformation.

**Figure 7:**
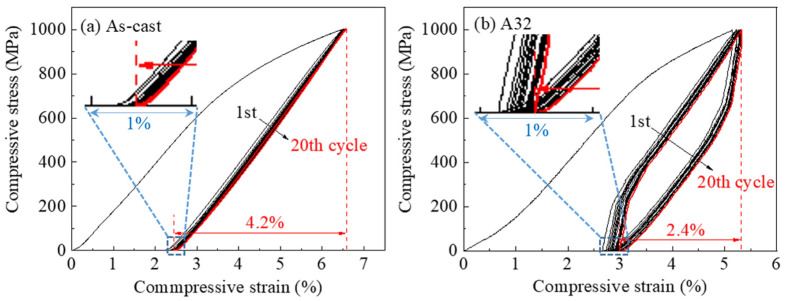
Cyclic deformation behaviour of (**a**) As-cast and (**b**) A32 samples at 298 K.

Figure 7 shows the cyclic compression stress–strain curves between 0 and 1000 MPa of the As-cast and A32 samples at 298 K. It is seen that the cyclic deformation behaviour is quite different for these two samples. Following the first unloading, the As-cast sample showed a large strain recovery of 4.3%. The subsequent loading-unloading cycles showed a quasi-linear stress–strain response and very lean stress hysteresis loop. The energy absorption of the 20th cycle is only 0.35 J/cm^3^. The drifts of the loaded strain (at 1000 MPa) and residual strain values (at 0 MPa) were very small, which appeared to stabilize after a few deformation cycles, suggestive of good fatigue performance. The shift of the residual strain following unloading can be seen in the inset in Figure 7a. The strain recovery of the 20th cycle is stabilized at 4.2% (marked in (a)). The cyclic deformation behaviour of A32 seems much less stable as compared with the As-cast sample. The hysteresis loop areas are quite large for all the cycles, with the energy dissipation values (18.35 J/cm^3^ of the 20th cycle) being more than 50 times greater than the As-cast sample. The deformation strain at 1000 MPa and residual strain (inset in Figure 7b) both showed a significant drift to larger values with the increase of deformation cycles. The recovery strain is much smaller being only 2.4% at the 20th cycle in sharp contrast to that of the As-cast sample.

The quasi-linear superelasticity and narrow stress–strain loop have been observed in nanostructured NiTi [39] and NiTi/Nb nanocomposites [40]. These phenomena are often related to the ultrafine grain size and prestrain under loading of the system. Wang et al. found that the prestrain process in a NiTi/Nb nanocomposite system can generate numerous dislocation loops at the NiTi/Nb interfaces, which gives rise to a broad distribution of internal local stress amplitudes and a broad distribution of remnant martensitic domains during loading [41]. They claimed that as long as the influence region of the local stress field can cover the whole region of the sample, the system will then show continuous growth of martensitic domains with quasi-linear superelasticity and small hysteresis under loading. In this sense, the dense grain boundaries/interfaces in either nanostructured single phase morphology or precipitated dual phase morphology can work as physical confinement to decrease space for martensitic transformation. In a dual phase system, the matrix phase deforms via martensite variant detwinning, while the precipitation phase deforms by dislocation slip. This means the interfaces could be the sites for producing dislocation loops (thus the internal stress field) due to the unmatched deformation mechanisms between the two phases. Therefore, the martensitic transformation starts in certain locations with different required stress levels and proceeds gradually as the applied stress increases. Apparently, among all the samples, the As-cast sample has the highest density of interfaces which can produce the most dislocation sources following prestrain and provide effective physical departmentalization to the Heusler matrix to suppress long range transformation strains. Following annealing at high temperature with long holding time (e.g., A32), the grain coarsening occurs and the density of interface decreases, leading to the disappearance of quasi-linear superelasticity and narrow hysteresis.

### 3.5. Crack Propagation and Fractographic Examination

**Figure 8:**
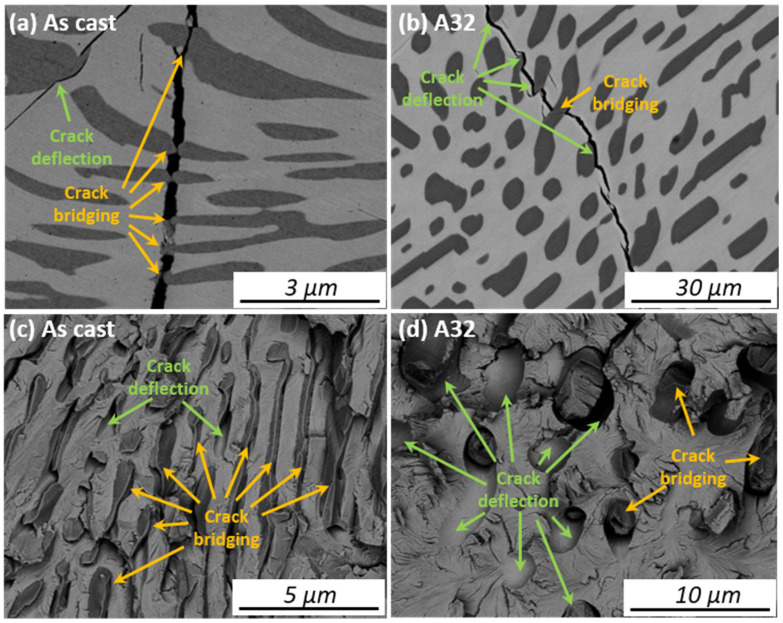
Crack propagation in different dual phase morphologies. (**a**,**c**): As-cast and (**b**,**d**): A32. All the micrographs are in the back-scattered electron mode.

It is well known that the purpose of the ductile second phase is to interact or interfere with the crack progression in ductile phase reinforced intermetallics. The main toughening mechanisms by the second phase are crack bridging and crack deflection [42,43]. With regard to the energy dissipation, the crack bridging mechanism contributes much more to the toughness of a component than the crack deflection mechanism because of the larger amount of plastic deformation involved. Figure 8 presents the back-scattered SEM micrographs that show the crack path and fracture features in the fine- and coarse-grained microstructures. Figure 8a shows the crack propagation in the As-cast alloy. The crack propagated through the γ phase lamellae, causing the bridging toughening. The γ phase grains showed clear necking (yellow arrows) and large plastic deformation via the interaction with the crack. In some part, crack deflection (the green arrow) is also seen but is considered only as a secondary toughening mechanism. Micrograph (c) is the fracture surface of the As-cast sample. One can see that the majority of the γ phase grains remain embedded in the Heusler matrix with a torn and ridge-like appearance. The Heusler matrix is effectively departmentalized by the finely spaced γ phase lamellae, making the passage for the transgranular cleavage cracking more difficult. Only a small number of the grain pull-outs can be seen, indicative of the crack deflection around the interface between the γ phase and the matrix.

Figure 8b shows the crack propagation in A32. Contrary to the situation in the As-cast, the cracks tend to deflect more around the γ phase grains rather than to penetrate through them. The fracture surface (micrographs (d)) also shows dominant interface debonding with a few torn-ruptured γ phase grains. Following annealing, the γ phase grains not only grow to larger sizes but also become more equiaxed in morphology. This makes the crack to deflect around them more easily because of the reduced average interface curvatures, given the same incident angle of a crack to the interface and same bonding strength of the interface. The above observation explains why the fracture strength and ductility of the fine grained As-cast alloy are higher compared to the coarse-grained ones.

## 4. Conclusions

The effects of isothermal annealing on the microstructure, martensitic transformation and mechanical properties were investigated in a Ni_51.5_Mn_34_Fe_6_Sn_8.5_ dual phase alloy. The experimental evidence and discussions lead to the following conclusions: 

(1) The fine lamellar eutectic microstructure of the Ni_51.5_Mn_34_Fe_6_Sn_8.5_ alloy coarsened progressively during isothermal annealing at 950 °C. The average grain size increased ten-fold after annealing for 120 h. 

(2) The volume fraction of the γ phase increased with the annealing time, as a result of the inter-diffusion of Fe and Sn between the Heusler matrix and the γ phase. The martensitic transformation temperatures, enthalpy and entropy changes decreased with the increase of annealing time due to the e/a ratio reduction in the Heusler phase.

(3) The thermomagnetic behaviour of the alloy was greatly affected by the isothermal annealing. The metamagnetic martensitic transformation was observed when annealed between 1 and 32 h. The As-cast sample did not show such characteristics as the transformation is largely above the Curie transition of the austenite.

(4) The compressive fracture strength and ductility decreased with the increase of annealing time. This is attributed to the increase of the grain size of the γ phase and change of the toughening mechanism from crack bridging dominant to crack deflection dominant. The best mechanical performance was found in the As-cast sample with a fracture strength of ~1845.0 MPa and ductility of ~22.6%.

(5) The cyclic deformation behaviour of the fine grained As-cast sample showed a quasi-linear superelasticity of 4.2% and narrow stress hysteresis loops, indicating the good fatigue performance. The coarse grained A32 sample showed a reduced superelasticity of 2.6% and degraded fatigue properties due to the large hysteresis loop areas and high energy dissipation under loading.

## Figures and Tables

**Table 1:** Composition, e/a ratio, Fe/Sn ratio of the matrix and γ phases for all samples.

Sample	Anneal Time (h)	Heusler Matrix						γ Phase					
		Ni (at%)	Mn (at%)	Sn (at%)	Fe (at%)	e/a	Fe/Sn	Ni (at%)	Mn (at%)	Fe (at%)	Sn (at%)	Ave Grain Size (μm)	Vol Fraction (%)
As-cast	0	49.7	35.5	11.0	3.8	8.199	0.345	53.2	34.0	10.5	2.3	0.31	28.8
A1	1	49.7	35.5	11.4	3.4	8.183	0.298	53.8	33.9	10.7	1.6	0.61	29.4
A2	2	49.6	34.8	12.4	3.2	8.148	0.258	54.2	33.1	11.2	1.5	0.72	30.6
A4	4	49.4	35.0	12.6	3.0	8.134	0.238	54.1	33.4	11.0	1.5	0.91	31.6
A8	8	49.4	34.8	12.8	3.0	8.128	0.234	53.9	33.9	10.6	1.6	1.17	32.7
A16	16	49.3	34.7	13.0	3.0	8.119	0.231	53.6	34.0	10.7	1.7	1.79	34.2
A32	32	49.4	34.5	13.2	2.9	8.115	0.220	53.9	33.9	10.7	1.5	3.10	35.3
A120	120	49.3	34.4	13.5	2.8	8.102	0.207	54.0	33.8	10.8	1.4	3.31	35.5

## Data Availability

The data presented in this study are available on request from the corresponding author.

## References

[1] Kainuma R., Imano Y., Ito W., Morito H., Sutou Y., Oikawa K., Fujita A., Ishida K., Okamoto S., Kitakami O. (2006). Metamagnetic shape memory effect in a Heusler-type Ni43Co7Mn39Sn11 polycrystalline alloy. Appl. Phys. Lett..

[2] Kainuma R., Imano Y., Ito W., Sutou Y., Morito H., Okamoto S., Kitakami O., Oikawa K., Fujita A., Kanomata T. (2006). Magnetic-field-inudced shape recovery by reverse phase transformation. Nature.

[3] Li Z., Jing C., Zhang H.L., Qiao Y.F., Cao S.X., Zhang J.C., Sun L. (2009). A considerable metamagnetic shape memory effect without any prestrain in Ni46Cu4Mn38Sn12 Heusler alloy. J. Appl. Phys..

[4] Gottschall T., Skokov K.P., Burriel R., Gutfleisch O. (2016). On the S(T) diagram of magnetocaloric materials with first-order transition: Kinetic and cyclic effects of Heusler alloys. Acta Mater..

[5] Krenke T., Duman E., Acet M., Wassermann E.F., Moya X., Manosa L., Planes A. (2005). Inverse magnetocaloric effect in ferromagnetic Ni-Mn-Sn alloys. Nat. Mater..

[6] Liu J., Woodcock T.G., Scheerbaum N., Gutfleisch O. (2009). Influence of annealing on magnetic field-induced structural transformation and magnetocaloric effect in Ni–Mn–In–Co ribbons. Acta Mater..

[7] Qu Y., Cong D., Sun X., Nie Z., Gui W., Li R., Ren Y., Wang Y. (2017). Giant and reversible room-temperature magnetocaloric effect in Ti-doped Ni-Co-Mn-Sn magnetic shape memory alloys. Acta Mater..

[8] Li Y., Sun W., Zhao D., Xu H., Liu J. (2017). An 8 K elastocaloric temperature change induced by 1.3% transformation strain in Ni44Mn45−Sn11Cu alloys. Scr. Mater..

[9] Shen Y., Wei Z., Sun W., Zhang Y., Liu E., Liu J. (2020). Large elastocaloric effect in directionally solidified all-d-metal Heusler metamagnetic shape memory alloys. Acta Mater..

[10] Ding Z., Zhu J., Zhang X., Liu D., Qi Q., Zhang Y., Cong D. (2016). 14% recoverable strain in Ni52.87Mn23.82Ga23.32 microwires. J. Phys. D Appl. Phys..

[11] Li F.Q., Qu Y.H., Yan H.L., Chen Z., Cong D.Y., Sun X.M., Li S.H., Wang Y.D. (2018). Giant tensile superelasticity originating from two-step phase transformation in a Ni-Mn-Sn-Fe magnetic microwire. Appl. Phys. Lett..

[12] Czaja P., Chulist R., Tokarski T., Czeppe T., Chumlyakov Y.I., Cesari E. (2018). Superelastic behavior of a metamagnetic Ni–Mn–Sn single crystal. J. Mater. Sci..

[13] Chernenko V., Villa E., Salazar D., Barandiaran J.M., Jaramillo D.S. (2016). Large tensile superelasticity from intermartensitic transformations in Ni49Mn28Ga23 single crystal. Appl. Phys. Lett..

[14] Chen Y., Schuh C.A. (2011). Size effects in shape memory alloy microwires. Acta Mater..

[15] Liu D., Cong D., Sun X., Chen H., Nie Z., Chen Z., Zhang Y., Zhu C., Qu Y., Zhu J. (2017). Low-hysteresis tensile superelasticity in a Ni-Co-Mn-Sn magnetic shape memory microwire. J. Alloy. Compd..

[16] Feng Y., Sui J., Gao Z., Zhang J., Cai W. (2009). Investigation on martensitic transformation behavior, microstructures and mechanical properties of Fe-doped Ni–Mn–In alloys. Mater. Sci. Eng. A.

[17] Feng Y., Sui J., Gao Z., Dong G., Cai W. (2009). Microstructure, phase transitions and mechanical properties of Ni50Mn34In16−yCoy alloys. J. Alloy. Compd..

[18] Ma Y., Yang S., Liu Y., Liu X. (2009). The ductility and shape-memory properties of Ni-Mn-Co-Ga high-temperature shape-memory alloys. Acta Mater..

[19] Ma Y.-Q., Lai S.-L., Yang S.-Y., Luo Y., Wang C.-P., Liu X.-J. (2011). Ni_56_Mn_25−x_Cr_x_Ga_19_ (x=0, 2, 4, 6) high temperature shape memory alloys. Trans. Nonferrous Met. Soc. China.

[20] Ma Y., Yang S., Jin W., Liu X. (2009). Ni_56_Mn_25−x_Cu_x_Ga_19_ (x=0, 1, 2, 4, 8) high-temperature shape-memory alloys. J. Alloy. Compd..

[21] Ma Y., Jiang C., Li Y., Xu H., Wang C., Liu X. (2007). Study of Ni_50+x_Mn_25_Ga_25−x_ (x=2–11) as high-temperature shape-memory alloys. Acta Mater..

[22] Yang S.Y., Liu Y., Wang C.P., Shi Z., Liu X.J. (2013). The mechanism clarification of Ni–Mn–Fe–Ga alloys with excellent and stable functional properties. J. Alloy. Compd..

[23] Yang S., Liu Y., Wang C., Liu X. (2012). Martensite stabilization and thermal cycling stability of two-phase NiMnGa-based high-temperature shape memory alloys. Acta Mater..

[24] Villa E., Villa E., Melzi D’Eril M., Nespoli A., Passaretti F. (2018). The role of γ-phase on the thermo-mechanical properties of NiMnGaFe alloys polycrystalline samples. J. Alloy. Compd..

[25] Yang S., Liu Y., Wang C., Lu Y., Wang J., Shi Z., Liu X. (2015). Microstructure and functional properties of two-phase Ni–Mn–Fe–In shape memory alloys with small transformation hysteresis width. J. Alloy. Compd..

[26] Yang S., Ma Y., Jiang H., Liu X. (2011). Microstructure and shape-memory characteristics of Ni56Mn25−xCoxGa19 (x = 4, 8) high-temperature shape-memory alloys. Intermetallics.

[27] Wu Z., Guo J., Liang Z., Zhang Y., Ye X., Zhang J., Li Y., Liu Y., Yang H. (2020). Room temperature metamagnetic transformation of a tough dual-phase Ni–Mn–Sn–Fe ferromagnetic shape memory alloy. J. Alloy. Compd..

[28] Wu Z., Liang Z., Zhang Y., Liu Z., Zhang J., Motazedian F., Bakhtiari S., Shariat B.S., Liu Y., Ren Y. (2019). A eutectic dual-phase design towards superior mechanical properties of heusler-type ferromagnetic shape memory alloys. Acta Mater..

[29] Ardell A.J. (1999). Microstructural stability at elevated temperatures. J. Eur. Ceram. Soc..

[30] Oriani R. (1964). Ostwald ripening of precipitates in solid matrices. Acta Met..

[31] Drolet J., Galibois A. (1968). The impurity-drag effect on grain growth. Acta Met..

[32] Hersent E., Marthinsen K., Nes E. (2014). On the Effect of Atoms in Solid Solution on Grain Growth Kinetics. Met. Mater. Trans. A.

[33] Abbaschian R., Abbaschian L., Reed-Hill R.E. (2010). Physical Metallurgy Principles.

[34] Park J.-H., Tomota Y., Wey M.-Y. (2002). Suppression of grain growth in dual phase steels. Mater. Sci. Technol..

[35] Krenke T., Moya X., Aksoy S., Acet M., Entel P., Manosa L., Planes A., Elerman Y., Yücel A., Wassermann E. (2007). Electronic aspects of the martensitic transition in Ni–Mn based Heusler alloys. J. Magn. Magn. Mater..

[36] Zheng H., Wang W., Xue S., Zhai Q., Frenzel J., Luo Z. (2013). Composition-dependent crystal structure and martensitic transformation in Heusler Ni–Mn–Sn alloys. Acta Mater..

[37] Koyama K., Watanabe K., Kanomata T., Kainuma R., Oikawa K., Ishida K. (2006). Observation of field-induced reverse transformation in ferromagnetic shape memory alloy Ni_50_Mn_36_Sn_14_. Appl. Phys. Lett..

[38] Zhang K., Tan C., Zhu J., Cai W., Guo E.J., Feng Z.C., Zhu J.C., Tong Y.X. (2018). Simultaneous tuning of martensitic transformation behavior, magnetic and mechanical properties in Ni–Mn–Sn magnetic alloy by Cu doping. J. Mater. Chem. C.

[39] Ahadi A., Sun Q. (2013). Stress hysteresis and temperature dependence of phase transition stress in nanostructured NiTi—Effects of grain size. Appl. Phys. Lett..

[40] Hao S., Cui L., Jiang D., Han X., Ren Y., Jiang J., Liu Y., Liu Z., Mao S., Wang Y. (2013). A Transforming Metal Nanocomposite with Large Elastic Strain, Low Modulus, and High Strength. Science.

[41] Wang D., Liang Q., Zhao S., Zhao P., Zhang T., Cui L., Wang Y. (2019). Phase field simulation of martensitic transformation in pre-strained nanocomposite shape memory alloys. Acta Mater..

[42] Evans A.G. (1990). Perspective on the Development of High-Toughness Ceramics. J. Am. Ceram. Soc..

[43] Evans A., Marshall D. (1989). The Mechanical Behavior of Ceramic Matrix Composites. Proceedings of the 7th International Conference on Fracture (ICF7).

